# Preliminary study of shark microbiota at a unique mix-species shark aggregation site, in the Eastern Mediterranean Sea

**DOI:** 10.3389/fmicb.2023.1027804

**Published:** 2023-02-23

**Authors:** Goni Bregman, Maya Lalzar, Leigh Livne, Eyal Bigal, Ziv Zemah-Shamir, Danny Morick, Dan Tchernov, Aviad Scheinin, Dalit Meron

**Affiliations:** ^1^Morris Kahn Marine Research Station, Department of Marine Biology, Leon H. Charney School of Marine Sciences, University of Haifa, Haifa, Israel; ^2^Bioinformatics Services Unit, University of Haifa, Haifa, Israel

**Keywords:** microbiome, shark, dusky, sandbar, bacterial profile, *Streptococcus* sp., biomarker, shark aggregation

## Abstract

Sharks, as apex predators, play an essential ecological role in shaping the marine food web and maintaining healthy and balanced marine ecosystems. Sharks are sensitive to environmental changes and anthropogenic pressure and demonstrate a clear and rapid response. This designates them a “keystone” or “sentinel” group that may describe the structure and function of the ecosystem. As a meta-organism, sharks offer selective niches (organs) for microorganisms that can provide benefits for their hosts. However, changes in the microbiota (due to physiological or environmental changes) can turn the symbiosis into a dysbiosis and may affect the physiology, immunity and ecology of the host. Although the importance of sharks within the ecosystem is well known, relatively few studies have focused on the microbiome aspect, especially with long-term sampling. Our study was conducted at a site of coastal development in Israel where a mixed-species shark aggregation (November–May) is observed. The aggregation includes two shark species, the dusky (*Carcharhinus obscurus*) and sandbar (*Carcharhinus plumbeus*) which segregate by sex (females and males, respectively). In order to characterize the bacterial profile and examine the physiological and ecological aspects, microbiome samples were collected from different organs (gills, skin, and cloaca) from both shark species over 3 years (sampling seasons: 2019, 2020, and 2021). The bacterial composition was significantly different between the shark individuals and the surrounding seawater and between the shark species. Additionally, differences were apparent between all the organs and the seawater, and between the skin and gills. The most dominant groups for both shark species were Flavobacteriaceae, Moraxellaceae, and Rhodobacteraceae. However, specific microbial biomarkers were also identified for each shark. An unexpected difference in the microbiome profile and diversity between the 2019–2020 and 2021 sampling seasons, revealed an increase in the potential pathogen *Streptococcus.* The fluctuations in the relative abundance of *Streptococcus* between the months of the third sampling season were also reflected in the seawater. Our study provides initial information on shark microbiome in the Eastern Mediterranean Sea. In addition, we demonstrated that these methods were also able to describe environmental episodes and the microbiome is a robust measure for long-term ecological research.

## Introduction

The Mediterranean Sea (MS) is the world’s largest and deepest enclosed sea. It is known as an oligotrophic sea with a decreasing gradient of nutrient concentrations from west to east (Lazzari et al., 2012). The biological diversity and productivity also follow this trend and are inversely related to the salinity and temperature gradients, which increase to the east (Moutin and Raimbault, 2002). The MS, nearly land-locked, is exposed to a growing human population and, as a result, an increase in pollution, resource exploitation, and anthropogenic activities in general. In addition, global climate change presence is reflected, *inter alia*, through rising water temperatures and salinities, which allows researchers to use the MS as a miniature model for the future of the world’s oceans (Lejeusne et al., 2010). Although the MS only consists of 0.82% of the world ocean area and 0.3% of the world’s oceans volume, and despite its unique characteristics, it is one of the significant reservoirs of marine and coastal biodiversity, containing 15,000–20,000 marine species, nearly a quarter of which are endemic (Coll et al., 2010; Lejeusne et al., 2010; Bianci et al., 2012). One of the key factors that enable the MS to sustain elaborate marine ecosystems is the existence of apex marine predators (AMPs). In the MS, 20 of the recorded 47 species of sharks are AMPs in coastal and pelagic ecosystems (Ferretti et al., 2008). Apex marine predators are crucial for maintaining healthy and balanced marine ecosystems by providing top-down regulation. Moreover, they play an important ecological role in shaping the marine food web by removing weak and sick organisms (Myers et al., 2007; Heithaus et al., 2008; Baum and Worm, 2009; Heupel et al., 2014). The sharks are sensitive to environmental changes (e.g., ocean acidification) and anthropogenic impacts (e.g., fishing and tourism intensity) and exhibit a clear and rapid response manifested in their physiological behavior and fitness (Skomal and Bernal, 2010; Hazen et al., 2019; Wheeler et al., 2020). In addition, sharks, maintain a K-selected life-history strategy (slow growth rate, late maturity, low fecundity, few offspring, long gestation periods, and long lifespans), making them extremely sensitive to population decline, particularly during vulnerable life stages (Ferretti et al., 2008; Hoffmayer et al., 2014). Studies from the last decade report that globally, the abundance of oceanic sharks has declined by 71% during the last 50 years (Bargnesi et al., 2020; Dulvy et al., 2021; Pacoureau et al., 2021). The MS is known for being one of the most dangerous places on Earth for sharks, including for chondrichthyan populations (although 26% of the species are considered data deficient by the IUCN; Ferretti et al., 2008).

Due to the significance of AMPs, including sharks (Raudino et al., 2016; Shamir et al., 2019), there is a strong argument for their use as a “keystone” or “sentinel” species. These species are organisms that indicate or respond to changes in the structure and functioning of the ecosystem and which, by monitoring, can yield essential ecological information (Hazen et al., 2019). Studies of sharks have focused on diverse aspects such as migration (Lawson et al., 2020), taxonomy (Vélez-Zuazo and Agnarsson, 2011; Elisio et al., 2019), physiology (Brooks et al., 2011; Elisio et al., 2019) and behavior (Klimley, 1994; Sims, 2010). However, only a few studies have examined sharks from a meta-organismal perspective and focused on the microbial aspect. This currently accepted approach considers the host and its associated microbiota as an inseparable functional unit (Zilber-Rosenberg and Rosenberg, 2008). The host offers favorable niches for microbes which, in turn, provide services to its hosts. These microbial niches change in their conditions, function and availability as the host organism develops and switches from one life-phase to the next (Williams et al., 2018; Gadoin et al., 2021). The diverse microbial communities can provide the host with optimal functionality and flexibility to changing environmental conditions (Zilber-Rosenberg and Rosenberg, 2008). On the other hand, a change in microbiota composition (due to physiological or environmental changes) can manifest as dysbiosis and may negatively affect the host (Rosenfeld, 2017). Many studies on marine organisms have examined the variation in microbiota in relation to its host species (Lima et al., 2012; Chiarello et al., 2017) life stage (Gadoin et al., 2021), health (Sweet and Bulling, 2017; West et al., 2019), ecology and environmental monitoring (Buck-Wiese et al., 2016; Glasl et al., 2019; Leray et al., 2021). These studies emphasize the importance of the microbiome, which can provide essential information on both the host and its environment. Few studies of shark microbiota have been published, the majority being in recent years. This paucity of data is a result of the complexity of capturing and sampling the shark while maintaining minimal injury to the animal. Indeed, researchers are constantly finding new, alternative sampling methods that are less harmful and non-invasive (Hammerschlag and Sulikowski, 2011). These studies have characterized the microbiome of different shark species (such as *Alopias vulpinus*, *Sphyrna tiburo* and *Triakis semifasciata*) from various geographic locations and are the first examples in this field (Doane et al., 2017, 2022; Leigh et al., 2021; Perry et al., 2021). In some cases, differences in the bacterial profile were seen between the shark species and the different organs (such as skin, gills, cloaca, and teeth; Caballero et al., 2020; Black et al., 2021). The difference in the bacterial profile was proposed, for example, to identify the species of shark in case of a shark bite, according to the bacteria that will be found in the wounds and also provide accurate medical treatment (Buck et al., 1984; Storo et al., 2021). Unfortunately, there is almost no information on infectious diseases or pathogens in sharks, except for a few reports of viral, bacterial, and fungal diseases, usually described as a disease case of a single shark (Terrell, 2004; Morick et al., 2020). Another study by Pogoreutz et al. (2019) compared the skin microbiome of healthy and injured black-tip reef sharks (*Carcharhinus melanopterus*). No difference was seen in the microbial community composition between the two cases. These results led to the assumption that the lack of infection or contamination in the injured shark’s skin due to its robust immune system (Pogoreutz et al., 2019).

The current study focuses on two species of shark found in the Eastern Mediterranean Sea (EMS), dusky (*Carcharhinus obscurus*) and sandbar sharks (*Carcharhinus plumbeus*). Both species are classified by the IUCN Red List: the dusky shark is “Endangered” (globally) and “Data Deficient” (Mediterranean Red List) and the sandbar is listed as “Vulnerable” (globally) and “Endangered” (Mediterranean Red List), with a decreasing population trend (IUCN, 2009). These species are considered large, cosmopolitan marine predators, undertaking long-distance migrations in coastal and pelagic waters (Rechisky and Wetherbee, 2003; Ferretti et al., 2008). During the last few decades, every year between November and May, about 40–80 individuals of these two *Carcharhinus* species aggregate very close to the shore, at the meeting point at a river estuary (Hadera stream) next to the outflows of a power and desalination plant (Barash et al., 2018; Zemah-Shamir et al., 2019). Interestingly, at this aggregation site, researchers observed that most of the dusky were females and most of the sandbar sharks are males (years 2015–2020; E. Bigal., *pers. comm.*, 22 August 2022; Zemah-Shamir et al., 2022a). Sex segregations in sharks, and other mixed groups, have been previously documented in different places around the world such leopard sharks (*T. semifasciata*; Nosal et al., 2013; Doane et al., 2022), *Carcharhinus amblyrhynchos* (Economakis and Lobel, 1998) and *Carcharodon carcharias* (Schilds et al., 2019). The aggregation can be of mixed groups (Speed et al., 2011; Jacoby et al., 2012), of one species and in some cases, of one gender (Nosal et al., 2013). Shark aggregations were observed in habitats with unique characteristics such as high temperature, high salinity, low water turbulence and high food availability (Hopkins and Cech, 2003; Hight and Lowe, 2007; Carlisle and Starr, 2009). The reasons for the aggregation are not yet fully understood, while various hypotheses such as mating, foraging, taking refuge, socializing and more have been proposed (Hight and Lowe, 2007; Speed et al., 2011; Nosal et al., 2013; Zemah-Shamir et al., 2022b).

The shark aggregation near the Israeli coast (Barash et al., 2018; Zemah-Shamir et al., 2022a) is localized and unique with consideration to its proximity to a coastal development site. First, the conditions at this site are associated with anthropogenic influences and includes warm temperature (5°C–10°C above the ambient temperature, all year long), increased salinity and organic matter enrichment (from the Hadera stream; Ministry of Environmental Protection, 2020). The second condition is the aspect of sex segregation and their seasonal aggregation to the site. Third, due to its accessibility and attractiveness, it is also exposed to heavy pressure from tourism, which affects the behavior of sharks (Zemah-Shamir et al., 2019). This specific aggregation allows annual monitoring (over the same months) of those shark species. Our study describes for the first time the microbiome of sharks in the EMS and provides an initial microbiome baseline. In addition, the study examines the use of the shark as a meta-organism, with an emphasis on its microbiome, as a tool that can reflect changes or environmental episodes.

## Methods

### Study site

The sampling aggregation site is located nearby the Orot Rabin power and desalination plant and near the Hadera Stream Estuary (32° 27′ N, 34° 52′ E) on the northern part of the Israeli coast (Supplementary Figure S1). The ambient seawater conditions in EMS range between 17°C and 27°C and 35–40 PSU. At the Hadera site, the temperature reaches up to 10°C and 5 PSU above the ambient seawater conditions year-round. The well-known aggregation occurs around the months of November to May and includes mainly adult female dusky sharks (*Carcharhinus obscurus*) and male sandbar sharks (*Carcharhinus plumbeus*) that are drawn to the plant’s warm effluents (Barash et al., 2018; Shamir et al., 2019). Therefore, November to May each year is defined here as a ‘sampling season’. Our sampling spanned November 2018 to May 2021, i.e., three sampling seasons (Supplementary Table S1).

### Shark sampling collection

Individual sharks were captured by using a handline or drumline and secured alongside the boat. Handling time was limited to 30 min, at which point the sharks were released safely back to the water. Microbiome samples were taken from three distinct anatomical locations (skin, gills, and cloaca) by gently rubbing a sterile swab (COPAN Diagnostics, United States) against the shark’s organs. In addition, an environmental sample (1.5-L surrounding seawater) was collected near each shark captured. The swabs and the seawater samples were kept sterile in a cool box until arrived at the laboratory. At the laboratory, environmental samples were filtered using a single use “Nalgene RapidFlow Filters” 0.2 μm (Thermo Scientific, cat no. 566–0020, Israel). All swabs and filtered environmental samples were stored at −20°C for further work. In total, 27 sharks (15 female dusky and 12 male sandbar sharks) were sampled, including 77 shark microbiome samples and 12 environmental samples (Supplementary Table S1). Mean ambient seawater temperature was measured in 10 m intervals by four acoustic receivers (Thelma Biotel, Norway) positioned around the power and desalination plant’s warm water plume.

### DNA extraction, PCR amplification, and amplicon sequencing

DNA was extracted from all samples (swabs and filters) using the DNeasy powerSoil Kit (Qiagen, Valencia, CA, United States) using the manufacturer’s instructions. PCR amplification of SSU rRNA gene fragments from the isolated DNA was done using universal primers (515F and 806R) as described in The Earth Microbiome Project (EMP; Stoeck et al., 2010; Caporaso et al., 2011) targeting the V4 regions of microbial small subunit ribosomal RNA genes. As described previously, the primers contained 5′ common sequence tags (CS1 and CS2; Moonsamy et al., 2013). Amplicons were generated using a two-stage PCR amplification protocol described by Naqib et al. (2018). Cycling conditions for the first stage PCR were 95°C for 5 min, followed by 28 cycles of 95°C for 30 s, 55°C for 45 s and 72°C for 60 s. Subsequent steps were carried out at Genome Research Core (GRC) within the Research Resources Center (RRC) at the University of Illinois at Chicago (UIC). Subsequently, a second amplification was performed for each sample, with a separate primer pair with a unique 10-base barcode obtained from the Access Array Barcode Library for Illumina (Fluidigm, South San Francisco, CA; Item# 100–4,876). Cycling conditions were 95°C for 5 min, followed by 8 cycles of 95°C for 30 s, 60°C for 30 s and 72°C for 30 s. Libraries were then pooled in equal volumes and the pool was purified using an AMPure XP cleanup protocol (0.6X, vol/vol; Agencourt, Beckmann-Coulter) to remove fragments smaller than 300 bp. The pooled libraries, with a 15% phiX spike-in, were loaded onto an Illumina MiniSeq mid-output flow cell (2 × 153 paired-end reads). Barcode sequences were used for sequence read de-multiplexing of raw data, which was then recovered as FASTQ-formatted files. The quality of the data was examined by FASTQC program and was found to be excellent for all samples, both directions of sequencing. Library preparation, pooling, and sequencing were performed at the Genome Research Core (GRC) within the Research Resources Center (RRC) at the University of Illinois at Chicago (UIC). Raw sequence data is available in the NCBI SRA database under BioProject ID PRJNA873249.

### Sequence data processing

The Dada2 pipeline^1^ (dada2 package version 1.18.0) was used for sequence data processing. Sequences were filtered and trimmed for quality using the “filterAndTrim” command with the parameters maxN set to zero, maxEE set to 2, trimLeft set to 20 bp and truncLen set to 153 bp for both forward and reverse reads. Sequence error estimation model was calculated using the “learnErrors” option using default parameters. Then, the dada2 algorithm for error correction was applied with the “dada” command using default parameters. Sequences were merged using the “mergePairs” command with a minimum overlap set at 8 bp. Each batch was processed separately up to this stage. Following, the three sequencing runs/batches were merged using Dada2 command “mergeSequenceTables.” Following, suspected chimera were detected and removed using the command “removeBimeraDenovo.” A count table including each amplicon sequence variant (ASV) in each sample was then produced. To obtain a taxonomic assignment for each ASV, each ASV sequence was aligned to the ARB-Silva small subunit rRNA database (version Silva_nr_138.1) using the command “assignTaxonomy” with default parameters, but minBoot set at 80%. A count table, adjoined with taxonomic assignment for each ASV was produced. All sequences with length < 247 bases or length > 252 bases were removed. In addition, ASVs of non-bacterial origin were filtered out (including chloroplast, mitochondria, Archaea and unclassified origin).

### Data analysis

#### Community structure

To examine community structure among shark species and/or organs, alpha-diversity parameters: Shannon H′ index of diversity, Simpson index, and species richness (represented by numbers of observed species, Sobs) were calculated and compared. These were calculated with “diversity” and “specnumber” commands in R package “vegan” (v2.5.7), using counts data normalized by subsampling to 23,500 (analysis of seasons 2019–2020) or 10,000 (analysis of season 2021) sequence reads per sample. The Shapiro–Wilk test performed rejected the null hypothesis of normality for all three indices for both shark species (*p* < 0.05). Therefore, statistical comparisons were conducted using the Kruskal–Wallis test. Differences were considered significant when *p* < 0.05. Where applicable, the *post hoc* pairwise Dunn test was performed (using R package FSA v0.9.3).

#### Community composition and similarity

To compare and examine the contribution of the factors species or organ to variation in microbiota composition, permutational analysis of variance (PERMANOVA) was performed (command “adonis” in R package “vegan”) based on Bray-Curtis dissimilarities, calculated using cumulative sum squares (CSS) normalized counts data. The permutations parameter was set to 999. Differences were considered significant for *p* < 0.05. Additionally, non-metric multidimensional scaling (NMDS) analysis was performed. NMDS was based on Bray-Curtis dissimilarities, calculated using CSS normalized counts data. NMDS was carried out using “metaMDS” command in R package “vegan” with parameters: k = 3 and try = 100. NMDS ordination was carried out a second time using counts data normalized to exclude the effect of the individual shark. For that, we applied the command “removeBatchEffect” from R package “limma” (v3.26.0) using shark ID as the “batch” factor and shark species and organ as factors in the design matrix. Following this normalization, NMDS was carried out again as described above.

In order to represent the composition of shark microbiota, CSS normalized counts were summed up to the genus, the family, the order and the class levels of taxonomy, and relative abundances were calculated (percent of total per sample). Stack-bars were drawn to include all taxonomic groups for which relative abundance was >3%. Linear discriminant analysis effect size (LEfSe) analysis was chosen to calculate differential abundance and identify biomarkers for shark species or organs. This method effectively determines which features, in this case ASVs, are most likely to explain observed differences among factor levels (Segata et al., 2011). LEfSe was performed using the online Galaxy module.^2^

#### Co-occurrence analysis

For the data obtained during 2021, co-occurrence analysis was performed. CSS normalized count data was summed up to the genus level. Then, genera which occurred in less than 20% of the samples (i.e., less than 8 samples) were filtered out. In total, 214 genera were retained. Following, Spearman correlations were pair-wise calculated and tested for significance. This was done using command “rcorr” in package “Hmisc” in R. *p*-values of the correlations were corrected for false discovery using the Benjamini–Hochberg correction (Supplementary Table S3).

#### Phylogenetic analysis of *Streptococcus*-affiliated ASVs

To examine relatedness between ASV sequences affiliated with the genus *Streptococcus* and others, including pathogenic, *Streptococcus* spp., a phylogenetic tree was calculated. This was done by alignment of all ASV sequences affiliated with the genus *Streptococcus* found in this study to the Arb-Silva small subunit ribosomal RNA database. The ACT engine of arb-silva web tools^3^ was used for alignment, and the 5 closest relatives from the databases for each ASV was curated along with ASV sequences. The alignment of ASV and relative sequences were used to calculate a phylogenetic tree (FastTree algorithm) in the ACT engine. The tree was visualized in FigTree.

## Results

Sharks were sampled during three sampling seasons between 2019 and 2021, near the Hadera power and desalination plants and the Hadera Stream Estuary (Supplementary Figure S1) on the northern part of the Israeli coast. Microbiota samples were collected from 15 dusky and 12 sandbar sharks, including three organs (skin, gills, and cloaca) and from the surrounding seawater, next to the caught shark (*n* = 89; Supplementary Table S1). In the sampling seasons 2019–2020, around 3,860,360 high-quality bacterial sequences (from 5,068,844 reads) were obtained, resulting in a total of 14,180 unique ASVs. Of these, 10,936 reads were identified to the Family level and 7,553 to the Genus level. For the dataset of 2021, 1,109,184 high-quality bacterial sequences (from 1,391,199 reads) were obtained, resulting in a total of 3,088 unique ASVs. Of these, 2,654 ASVs were identified to the Family level and 1,892 ASVs to the Genus level.

### Composition and diversity of the shark microbiota

**Figure 1:**
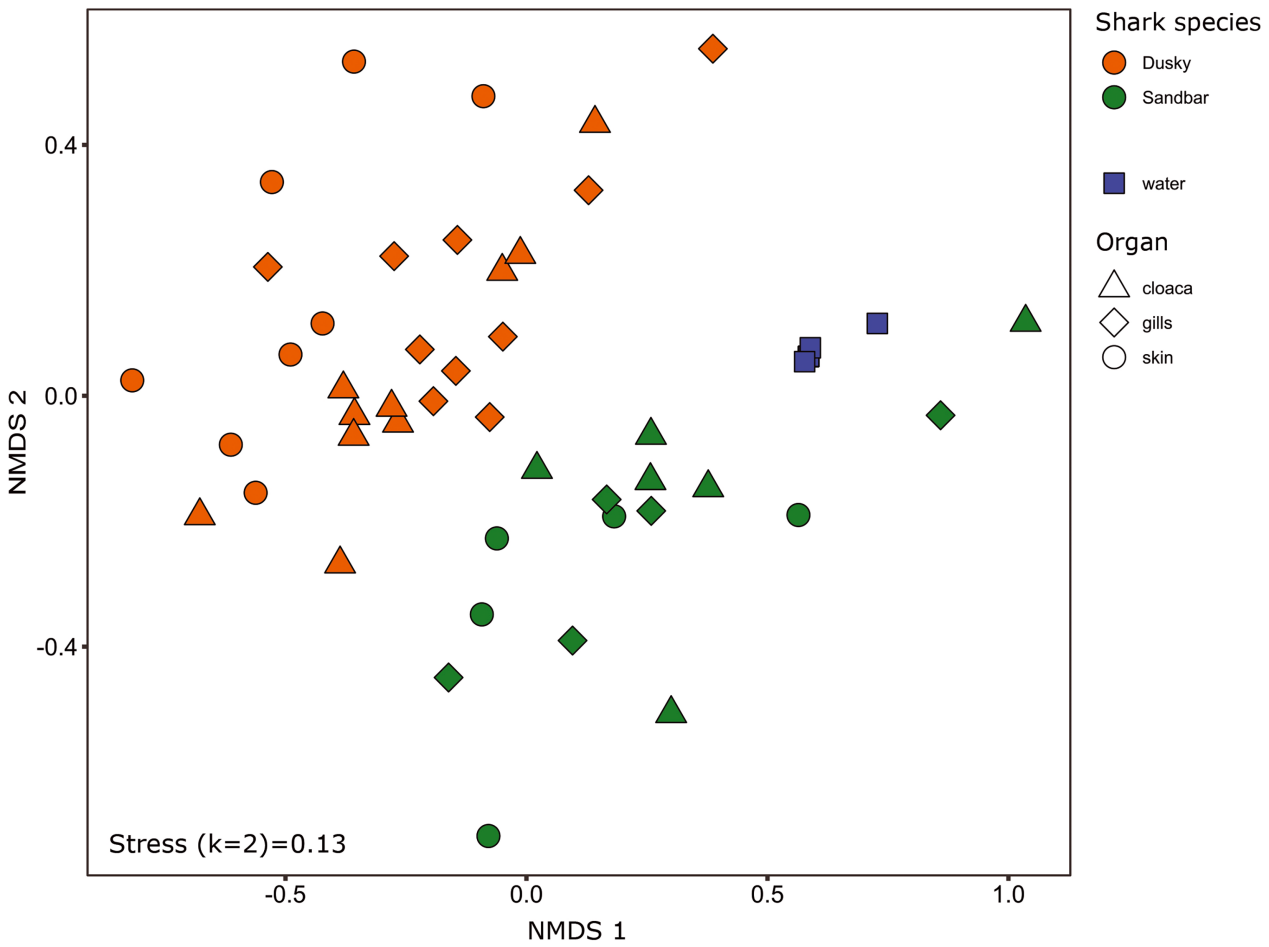
Bacterial profiles of shark species, organs and the surrounding seawater. Non-metric multidimensional scaling analysis was calculated based on Bray–Curtis dissimilarities among samples. Colors represent shark species, and shapes represent different organs.

**Figure 2:**
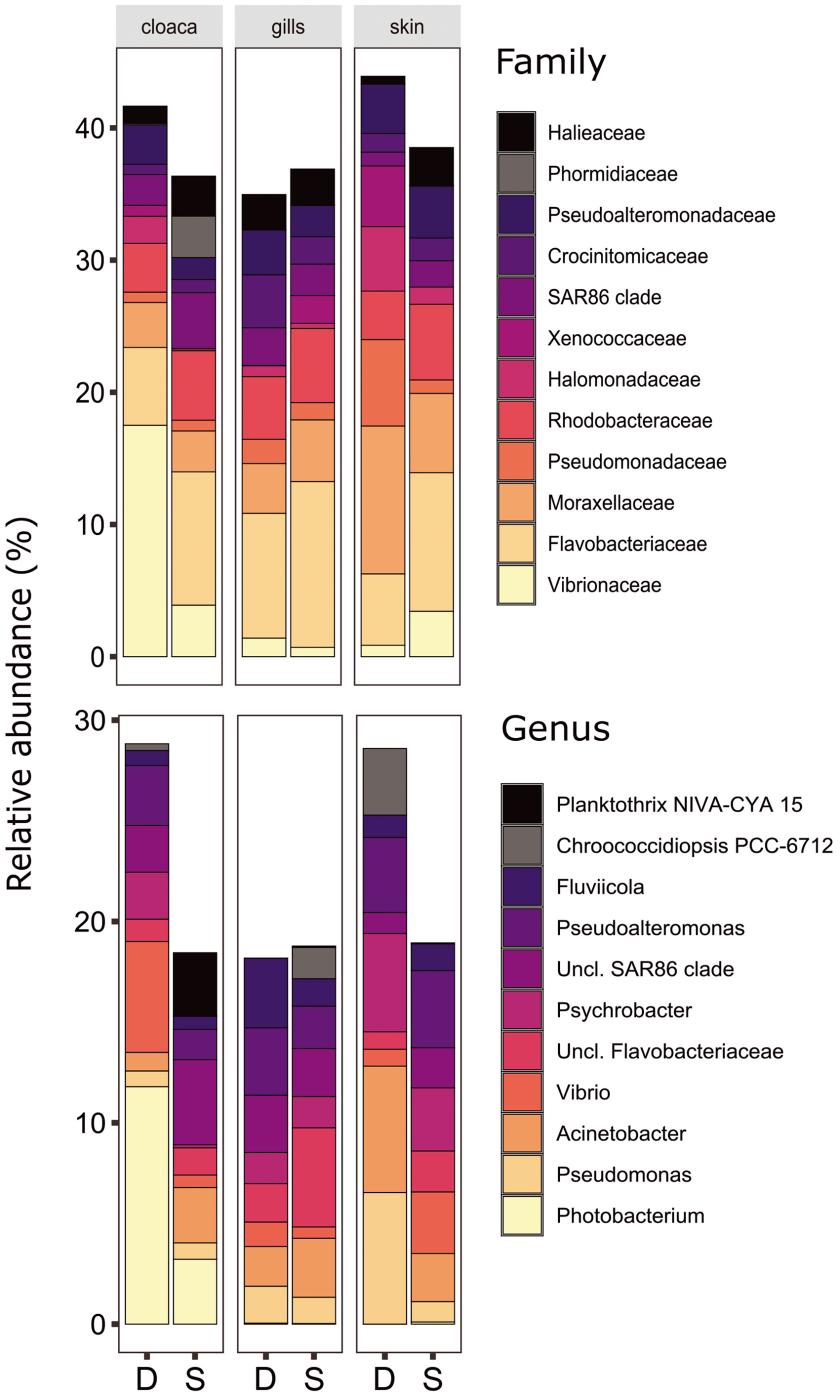
Bacterial compositions and relative abundance to Family (top) and Genera (bottom) resolution of dusky (D) and sandbar (S) sharks across three distinct organs. All groups below 3% relative abundance were not included.

The microbiota samples were compared and analyzed by NMDS to examine the factors that influence the microbial profile of sharks. The individual shark was the main contributing factor affected the microbiome composition (R2 = 0.12; *p* = 0.001). In addition, the seawater samples were clustered separately and were significantly different from shark samples (dusky: R2 = 0.18; *p* = 0.049, sandbar: R2 = 0.12; *p* = 0.002; Supplementary Figure S2). Follow-up analysis after neutralizing the individual effect (see Methods) showed a distinct difference between shark species (Figure 1; Supplementary Table S2). The bacterial composition of the environmental samples (surrounding seawater) was similar and clustered separately. The PERMANOVA test showed a significant effect of the organs (*p* = 0.018) and the *post hoc* pairwise Dunn tests accurately pinpointed the differences. Significant differences were shown between all organs and the seawater (*p* = 0.001), however among the organs only skin vs. gills exhibited a significant difference (*p* = 0.039; Supplementary Table S2). The microbiota composition of each of the shark species and organs was characterized by family and genus taxonomic levels (Figure 2). The most dominant family groups in both shark species and across all anatomic locations were Flavobacteriaceae, Moraxellaceae and Rhodobacteraceae. Examination of each shark species separately revealed a distinct microbiota composition for each of the organ. The major difference was observed in the cloaca bacterial of dusky shark, which was characterized mainly by Vibrionaceae (17% vs. less than 4% in the entire samples), which included the genera *Photobacterium* (12%) and *Vibrio* (5%). The microbiome of dusky skin consists of relatively high levels of the genera *Pseudomonas* and *Acinetobacter*. Additional groups identified as distinctive belonged to cyanobacteria. The family Phormidiaceae (including the *planktothrix NIVA-CYA 15*) was found in the sandbar‘s cloaca, while the family Xenococcaceae and the genus *Chroococcidiopsis PCC-6712* were found in the skin and gills of dusky sharks. These groups were absent or appeared at very low percentages in the other samples.

Alpha diversity indices, including Shannon H′, Simpson and richness (number of ASVs), were calculated and compared to assess the differences in community structure between samples (Supplementary Figure S3). The Kruskal–Wallis test indicated significant differences among dusky sharks, sandbar sharks and seawater for both Shannon H′ (χ2 = 10 df = 2, *p* = 0.006) and Simpson indexes (χ2 = 8.48, df = 2, *p* = 0.01). The *Post-hoc* Dunn test confirmed that the Shannon H′ and Simpson indexes were higher for sandbar sharks compared to dusky sharks (*p* = 0.02 and *p* = 0.04) or seawater (*p* = 0.02 and *p* = 0.03; Shannon H′ and Simpson indices respectively). When comparing the diversity indices between the anatomic locations, significant differences were observed only for the dusky shark (Shannon H′: χ2 = 8.06, df = 2, *p* = 0.017 and Simpson: χ2 = 8.2762, df = 2, *p* = 0.015), while the gills were the most diverse organ, particularly in comparison to the cloaca (Shannon H′: *p* = 0.027; Simpson H′: *p* = 0.03) and to the skin (Simpson H′: *p* = 0.03).

**Figure 3:**
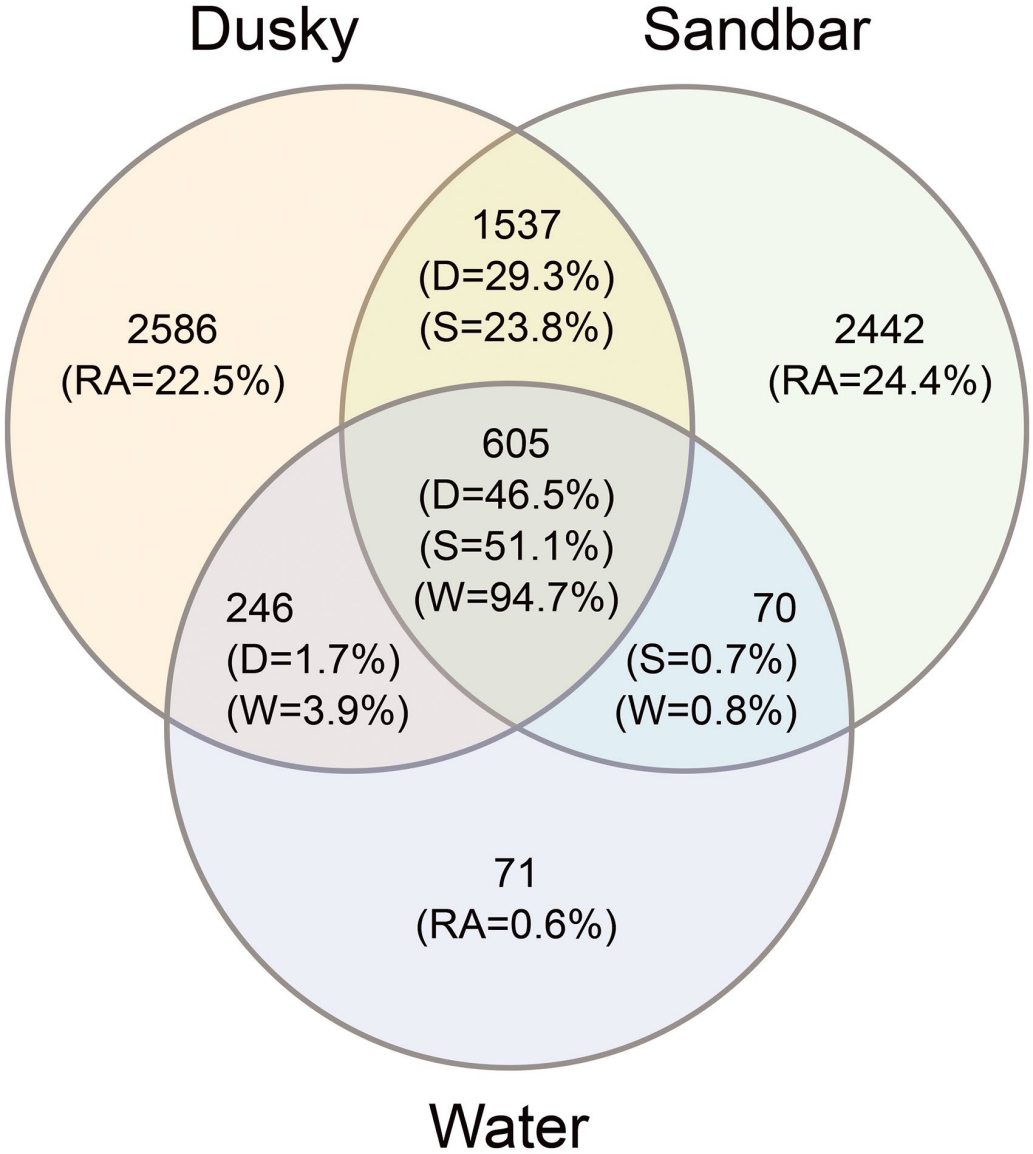
Venn diagram showing the number and relative abundance (RA) of unique and shared ASV’s among dusky sharks (D), sandbar sharks (S) and their surrounding seawater (W).

In order to examine the effect of the environmental microbiota on the bacterial profile of sharks, the number and relative abundance of the shared and unique ASVs among sharks and their surrounding seawater were calculated (Figure 3). The total ASVs for both sharks (sandbar 4,654 and dusky 4,974) were 4–5 times higher than that of seawater (992). Around 22%–24% of the shark’s ASVs were unique, while 23% and 29% (dusky and sandbar, respectively) were shared between the sharks. In contrast, only 0.6% of the seawater ASVs were unique to the seawater, and low percentages of the sequences were common with each of the shark species (3.9% with dusky and 0.8% with sandbar). Interestingly, most of the seawater ASVs (94.7%), dusky (46.5%) and sandbar (51.1%) were common for all three categories.

**Figure 4:**
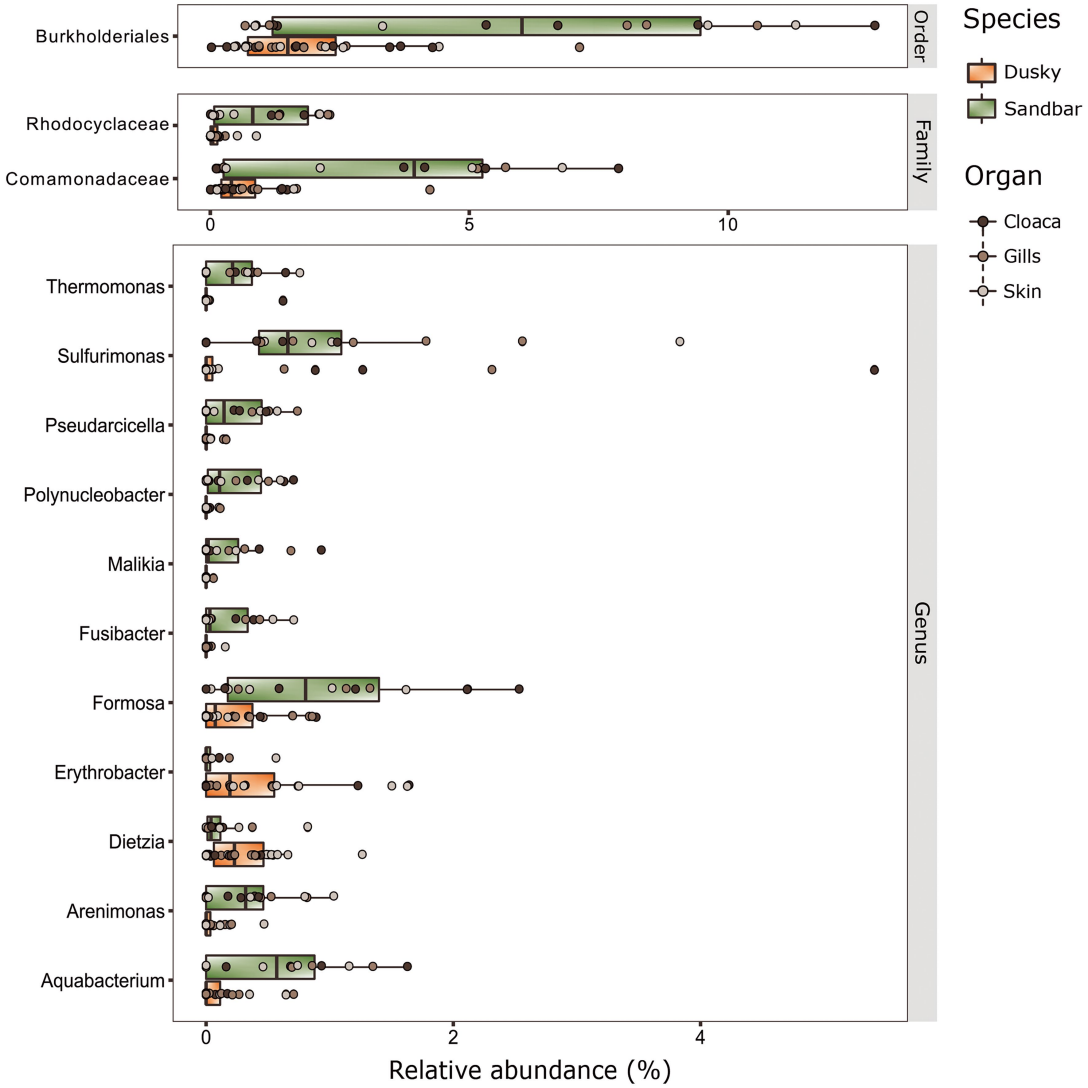
Biomarkers at the taxonomy levels (Order, Family and Genus) of dusky and sandbar sharks across three distinct anatomic locations were identified by the linear discriminant analysis effect size (LEfSe) methods (adjusted *p* < 0.05, LDA > 2).

Distinctive features were searched among shark species and organs, using a LEfSe test (LDA effect size) run across all taxonomic levels. A few biomarkers were identified (adj. *p* < 0.05, LDA score > 2.0) at the order, Family, and mostly at Genus taxonomic levels (Figure 4). Most of the biomarkers were assigned to sandbar sharks and found in very low relative abundance in the dusky sharks. The order Burkholderiales and the families Comamonadaceae and Rhodocyclaceae (of order Burkholderiales) were the only three biomarkers identified at these taxonomic levels and were significantly higher in sandbar sharks. The most dominant biomarkers of the sandbar, at the genus level, were *Aquabacterium*, *Formosa* and *Sulfurimonas*, while the *Erythrobacter* and *Dietzia* were linked to dusky sharks.

### Variation between the sampling seasons

**Figure 5:**
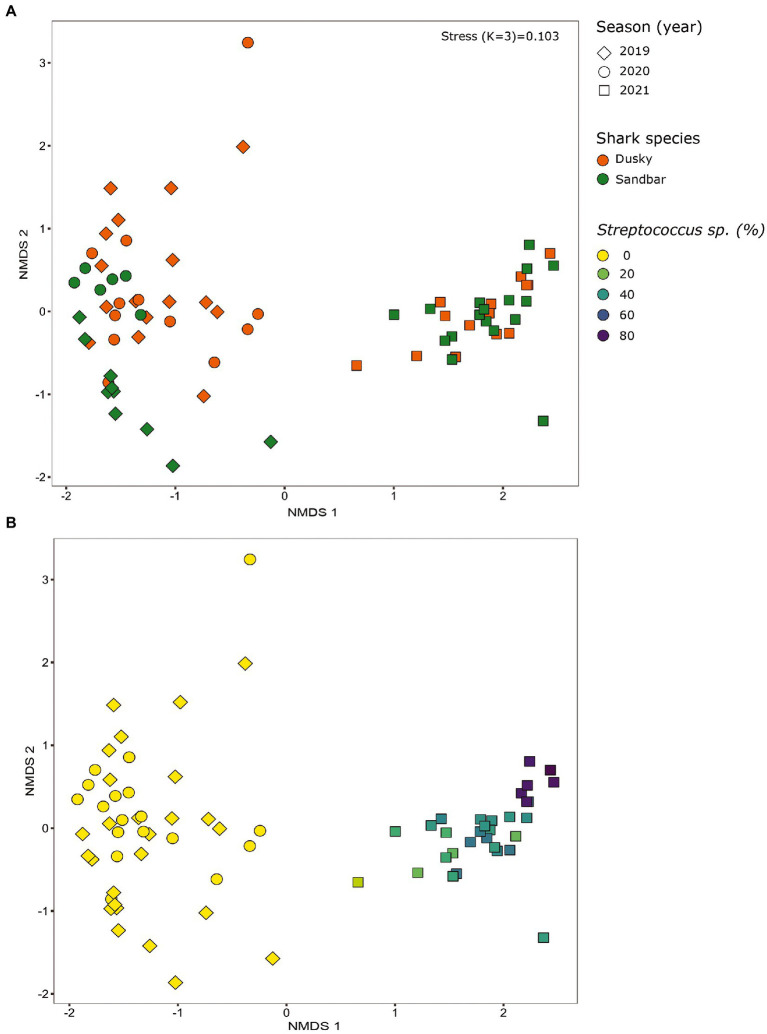
Bacterial profiles of sharks across the seasons (years). Non-metric multidimensional scaling analysis was calculated based on Bray-Curtis dissimilarities among samples. The figures show the distribution of the samples by shark species **(A)** and by the percentage of *streptococcus* sp. in each sample **(B)**.

**Figure 6:**
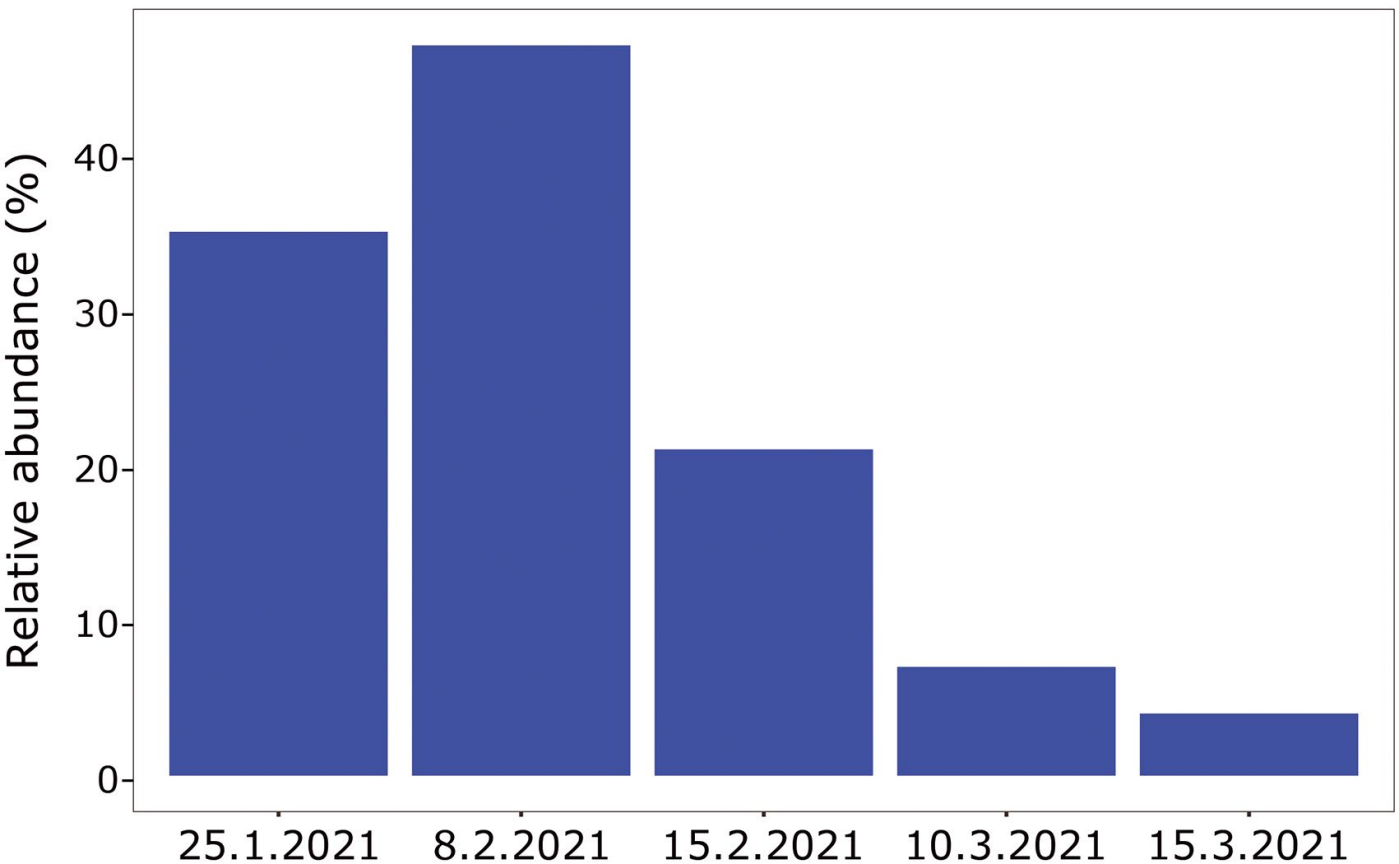
Relative abundance of *Streptococcus* sp. isolated from surrounding seawater samples, season 2021.

**Figure 7:**
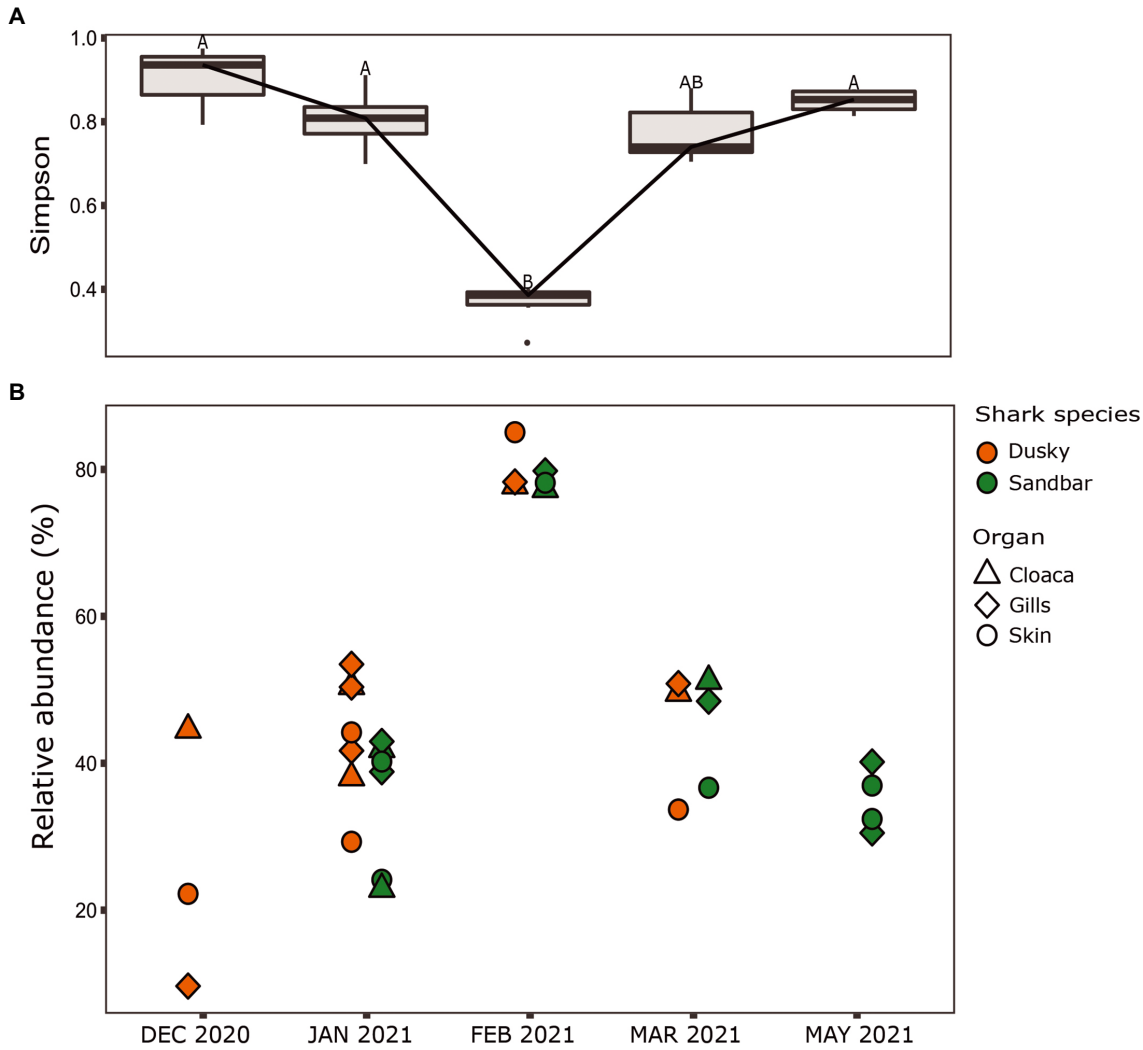
Season 2021. **(A)** Simpson diversity index of sharks’ samples. The letters A/B above the boxes represents a significant difference between the samples. **(B)** Relative abundance of *Streptococcus* sp. isolated from the shark species.

When comparing the shark samples of all three sampling seasons, a significantly different bacterial profile was obtained between the first seasons (2019–2020) and the third season (2021). In contrast to the previous seasons, Figure 5A shows a distinctly different cluster of 2021 season, which includes both shark species together. Analysis of the microbial community composition of the last season showed a high and abnormal presence of one specific ASV (ASV0002), which contributed to the differences of the previous seasons. This ASV was identified, according to NCBI, as *Streptococcus* sp. (similarity of 100%), and was found in all 2021 samples and their presence ranged from 10 to 85%. For comparison, this ASV was found only in two samples in previous seasons (one sample from each species of shark) and negligible percentages (less than 1.5%; Figure 5B). Examination of the presence of ASV0002 in seawater exhibited a similar picture. While in previous seasons, this variant was absent from the seawater samples, in the 2021 season, it was found from January to March in the range of 4%–35% of the bacterial community (Figure 6). Due to the high season variability and dominance of *Streptococcus* sp., this season was analyzed separately. In contrast to the previous seasons, no significant differences were detected between shark species and the organs. However, analysis of the samples showed that despite the dominance of the *Streptococcus* sp. in the sharks and seawater samples, a difference was observed in the relative abundance and the diversity (Simpson index) between the months of the season (December–May; Figures 6, 7A,B). The average Simpson index score for every month demonstrates a downward trend in Simpson values from December (0.92) to February (0.4) and an upward trend back from February to May (0.84; Figure 7A). This trend was reflected in the inverse correlation of *Streptococcus* sp. (ASV0002) percentages in shark microbiome samples (both species) over those months. Low percentages were detected in December (average 25%), peaked in February (average 79%), and declined back in May (average 35%; Figure 7B). Interestingly, when examining the presence of the *Streptococcus* sp. (ASV0002) in the surrounding seawater in this season, a similar trend to that in sharks samples was seen, increasing until its peak in early February and a declining after that (Figure 6).

**Figure 8:**
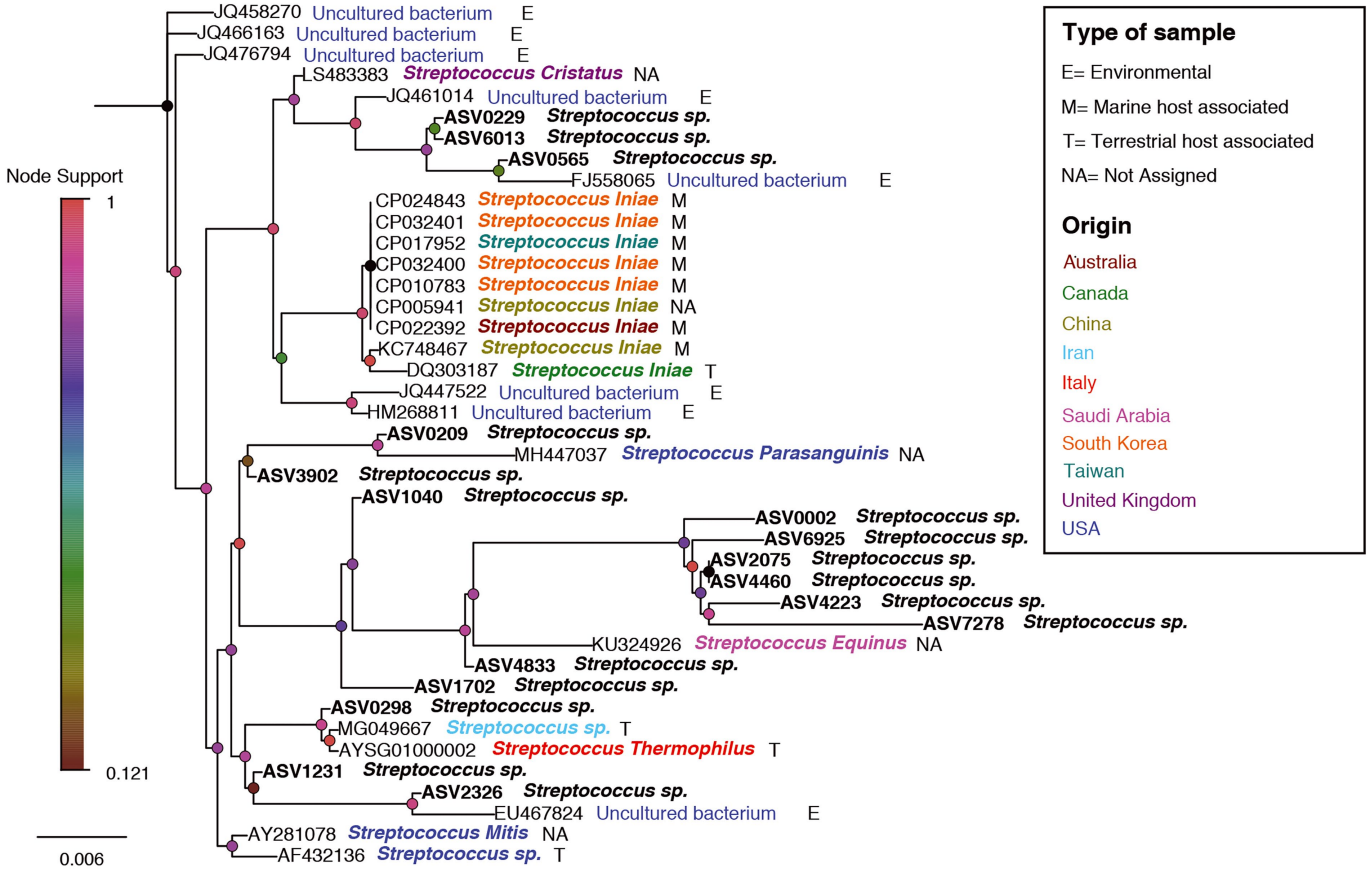
A phylogenetic tree for *Streptococcus*-related ASVs and accessions no. from NCBI. ASV0002 is marked by a red arrow. Nodes are colored according to the node-support bar. The taxonomy, next to ASV/Accession No., is colored according to the geographic origin while the letters (E/M/T/NA) represent the type of sample, according to the attached legend.

In order to examine relation between sequences affiliated with the genus *Streptococcus*, found in this study with sequences from other studies, a phylogenetic tree was designed. The phylogenetic tree provided information about the geographical origin and the type of sample (environmental, marine/terrestrial host-associated) from which the sequences were sampled (Figure 8). The phylogenetic tree showed clusters from different geographical origins, such as from the United States (primarily from environmental samples) and the Far East (including Taiwan, South Korea and China). The sequences from the Far East cluster, were mostly sequences associated with the marine host and identified as *Streptococcus inia*, a known marine pathogen. Interestingly, most of the sequences from our study, including ASV0002, were closer to related sequences from terrestrial hosts and from geographical origins (Saudi Arabia, Iran, and Italy), which are relatively close to our study area (Mediterranean region). To evaluate the effect of an increase in *Streptococcus* sp. in the shark’s microbiome, the abundance of the identified dominant bacteria from the 2019–2020 sampling seasons was compared to the 2021 season (Table 1). An increase of potentially pathogenic bacteria (*Photobacterium*, *Vibrio*, *Acinetobacter* sp.) were observed almost in all the samples, both shark species and especially in the gills of the dusky shark. In addition, a sharp increase was detected in the bacteria known as shark’s symbiont (*Pseudoalteromonas* and *Pseudomonas* sp.) across all samples, with higher values in sandbar sharks.

**Table 1:** Comparison of dominant bacteria between 2019 and 2020 seasons and the season 2021.

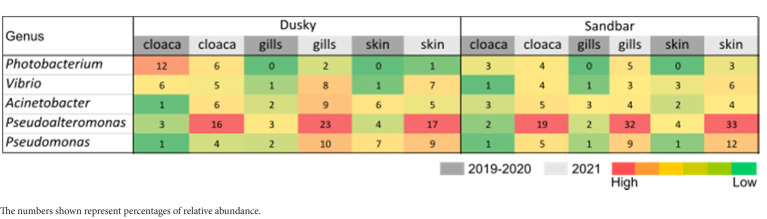

The numbers shown represent percentages of relative abundance.

We further examined whether co-occurrence patterns can be detected between *Streptococcus* and other genera in 2021, where that genus was dominant. In total, 59 significant Spearman correlations (FDR adjusted *p* < 0.05) were identified, of which only seven were positive correlations (Supplementary Table S3). The top groups (five positive and five negative) presented in Supplementary Figure S5.

## Discussion

Our study focused on the local aggregation of two shark species (male sandbar sharks and female dusky sharks). The aggregation site is a disturbed site that exposes the sharks to distinctive environmental conditions and high anthropogenic activity. This local phenomenon gave us a rare opportunity to conduct prolonged sampling of the shark community, emphasizing the microbiome aspect.

### Drivers of the sharks’ microbiota composition

Microbiota samples of skin, gills, and cloaca of both shark species and a water sample concurrent to the sampling time were collected. As these samples were derived from wild species *in situ*, after being caught and brought alongside the vessel, our study required this additional comparative analysis as extra assurance that the communities were significantly distinct. Comparison of the microbiota composition between the seawater and shark was significantly different in composition (Supplementary Figure S2; Supplementary Table S2) and reinforces the reliability of this non-invasive method, as it was impossible to isolate the sampling from the seawater. Our study showed that the main driver that effected the sharks’ microbiome was the specific host, i.e., the individual shark examined (Supplementary Figure S2). This finding corresponds with other microbiome studies conducted on fish, dolphins (Lima et al., 2012; Chiarello et al., 2017) and humans (Turnbaugh et al., 2007; Gilbert et al., 2018). Moreover, this individual factor is affected by the gender of the organism (Cabal et al., 2018) and its genetic material (Opstal and Bordenstein, 2015). In addition, the physiological condition of the organism, such as disease (Cho and Blaser, 2012), pregnancy (Neuman and Koren, 2017), metabolic state (Bang et al., 2018) and developmental phase (Gadoin et al., 2021) may affect and shape the composition of the microbiota. Interestingly, differences in the cohort’s behavior have been reported in previous studies to affect the microbiome (Archie and Tung, 2015; Vuong et al., 2017). For sharks, shifts in migration patterns (residency, depth profiles) and behavior under duress or related to reproduction (mating, gestational cycle) all may be impacted the microbiome. Following neutralization of the individual effect, additional drivers (shark specie and organ) shaping the microbiome were obtained. A significant division was shown between the sharks (dusky and sandbar) and between each shark to their environmental samples (Figure 1; Supplementary Table S2). Species-specific microbiomes have been reported in previous studies that compared three (Black et al., 2021) and five (Storo et al., 2021) shark species. The species-specific microbiomes were also observed in other wild apex predators such as bottlenose dolphins (*Tursiops truncatus*, *Tursiops aduncus*), sperm whales (*Physeter macrocephalus*; Erwin et al., 2017) and killer whales (*Orcinus orca*; Lima et al., 2012; Chiarello et al., 2017). Despite the difference between the sharks and the surrounding water, environmental conditions are known to influence the composition of the microbiota. Many studies investigated various environmental parameters such as pH, salinity, and temperature and demonstrated a shift in the host-microbial community due to changes in these conditions (Schmidt et al., 2015; Lima et al., 2020; Posadas et al., 2022). Although this site is characterized by high temperature, we could not isolate the direct effect of temperature on the composition of the microbiome, due to the lack of additional site for comparison. In addition, no substantial changes in temperature were observed between the sampling seasons (Supplementary Figure S4), so we could not attribute the observed differences between the seasons to the temperature factor. In this study, a clear overlap of ASVs was observed between the sharks (both species) and the seawater. About 50% of the shark’s ASVs were also found in the seawater samples, while the rest, ~25%, overlapped between the sharks and about ~25% of ASVs were unique to the shark species (Figure 3). Similar percentages of overlap sequences between sharks and seawater have been described in Storo et al. (2021).

The organs of the host serve as a separate micro-habitat. The organs are exposed to different levels of the environment and include unique conditions, such as physical properties, salinity, pH, temperature, and oxygen, which allows different microbial communities to inhabit them (Costello et al., 2009). Many studies examined and compared the microbiome between multiple organs (internal and external) of marine organisms, including fish (Meron et al., 2020; Sylvain et al., 2020), sea turtles (McNally et al., 2021) and mussels (Musella et al., 2020). Of the studies conducted on shark microbiome, most focused on one anatomic organ, such as skin and its associated mucus layer (Doane et al., 2017, 2022; Pogoreutz et al., 2019; Caballero et al., 2020) or gut (Givens et al., 2015; Juste-Poinapen et al., 2019; Leigh et al., 2021), while only a few studies compared between multiple anatomic locations as gills, teeth, skin, and cloaca (Black et al., 2021; Perry et al., 2021; Storo et al., 2021). Similar to our results, differences in microbiota between the organs were observed, some of which were statistically significant (depending on the of shark species and organ).

### Microbial diversity and core taxa

The diversity index indicates higher diversity in sandbar sharks than dusky sharks and their environment. Among the shark organs, the gills were more diverse, as observed by Storo et al. (2021) as well. The gills have numerous crucial functions, as a primary site for gas and waste exchange, as well as for mucosal immune interactions, osmoregulation, detoxification and possibly ammonia recycling (Wood et al., 2007; Pratte et al., 2018; Perry et al., 2021). The ability of elasmobranchs to absorb ammonia through the gills may help renew the urea used in regulating osmosis (Nawata et al., 2015; Wood and Giacomin, 2016). Therefore, gill-associated microbes may play critical roles in the overall fish and shark health and physiology, which may explain the gill’s increased diversity and richness (Supplementary Figure S3).

The number of studies that have described the bacterial profile of sharks is relatively small and makes the comparison difficult. They include different shark species, diverse organs, and varying geographical locations, as described in the review by Perry et al. (2021). Our study is the first research on Carcharhinidae microbiome yet in the Mediterranean and provides an initial baseline on the bacterial profile of the sharks at a highly localized site. However, we still found several taxonomic groups of bacteria in the same organs of different shark species described in previous studies. For example, the families Rhodobacteraceae and Halomonadaceae and the genera *Pseudoalteromonas* and *Psychrobacter* were dominant in the skin microbiota of our sharks but were also observed in the skin of thresher shark (*A. vulpinus*) and black-tip reef sharks (*C. melanopterus*; Doane et al., 2017; Pogoreutz et al., 2019). *Psychrobacter*, which was previously identified also in the skin mucus of bony fish (Juni, 1992) and humpback whale, have been linked with whale health and immunity (Apprill et al., 2014; Bierlich et al., 2018). The presence of *Pseudoalteromonas* has been described as a common symbiont on the skin of sharks that may contribute to the synthesis of antibacterial compounds and inhibit the growth of biological contamination on the skin of sharks (Holmström and Kjelleberg, 1999; Franks et al., 2006; Offret et al., 2016). Additionally, the genus *Pseudomonas* that was dominant in our study, mainly in the skin of dusky sharks (females), was found in the epidermal microbiome of leopard sharks (*T. semifasciata*), notable females of this species as well (Doane et al., 2022). Currently, there are not enough studies describing the microbiome of males vs. females, especially of the same shark species. However, future expansion of the database together with additional physiological parameters may enable the microbiome to reflect, among other things, gender, reproductive status, etc., through relatively simple sampling and a non-invasive technique. Another dominant bacterium in our skin samples and not reported in previous skin shark studies was the genus *Acinetobacter*. These bacteria can survive in various environments containing small amounts of nutritious components and display a low sensitivity to adverse physical and chemical conditions (Kozińska et al., 2014). These traits may have an advantage in areas of high temperature and salinity, which could possibly explain the presence at the studied site. However, more research is needed to better understand this aspect. The genera *Photobacterium* and *Vibrio* (both belonging to Vibrionaceae) are known as “common” bacteria in marine organisms, as part of their natural microbiota composition (Ramaiah and Chandramohan, 1992; Urbanczyk et al., 2011) or as potential pathogens (Buck, 1990; Terceti et al., 2016). In addition, the appearance and increase of *Vibrio* in marine organisms have been linked to an increase in the ambient temperature (Vezzulli et al., 2009; Kimes et al., 2012), which is the main characteristic of our study site. Although no signs of disease were seen in our study, these genera were the most dominant in the cloaca, specifically in dusky sharks. Based on previous studies in which the *Photobacterium* has been identified in the shark’s gastrointestinal tract (i.e., gut and cloaca; Grimes et al., 1985; Givens et al., 2015; Juste-Poinapen et al., 2019; Leigh et al., 2021), it has been suggested that it may be an integral part of the core intestinal microbiome of sharks (Perry et al., 2021).

### Bacterial biomarkers

Identification and use of bacterial biomarkers can be expressed *via* two aspects. The first is their use as an efficient detection tool of a specific organism, by the presence and relative abundance of specific bacterial taxa groups. In our case study, we identified a few bacterial biomarkers for shark species and organs by calculating the differential abundance of the ASVs. Most of the biomarkers linked to the sandbar sharks were significantly higher than the dusky sharks. Interestingly, the most dominant biomarkers belong to the class Betaproteobacteria (phylum Pseudomonadota) and were identified across a few taxonomic levels [e.g., Burkholderiales (Order) > Comamonadaceae (Family) > *Malikia* (Genus); Burkholderiales (Order) > Comamonadaceae (Family) > *Aquabacterium* (Genus)]. Future comparison between additional variables such as gender, physiological state, developmental stage, etc., will enable the expansion and refinement of additional biomarkers. The second aspect is the use of bacterial biomarkers as a tool for defining ecosystem health and stability due to the bacteria’s sensitivity and rapid response to environmental changes. Various marine organisms, mainly sessile organisms such as oysters and ascidians, have been proposed as potential bioindicators for environmental health, including monitoring of heavy metals (Huang et al., 2007; Tzafriri-Milo et al., 2019), organic enrichment (Babaranti et al., 2019) or drugs (Navon et al., 2020). However, the focus was on organism survival, its physiology, and tissue accumulation. In recent years, there has been an increase in research on the marine microbiome and the possibilities of using it as a bioindicator for environment health. These studies focused on the environmental microbiome of seawater and sediment (Chen et al., 2019; Custodio et al., 2022) but also the host-microbiome, as part of the holobiont. The body of literature to date includes mainly studies on invertebrates such as sponges (Selvin et al., 2009), corals (Ainsworth and Gates, 2016; Becker et al., 2022) and oysters (Maruf Billah and Rahman, 2021), and less in nekton such as fish and Chondrichthyes. No references were found in the literature regarding biomarkers of dusky or sandbar sharks and, to the best of our knowledge, this is the first study to cover this aspect.

### Dysbiosis

Our sampling in 2021 was the third occasion where samples from the same season, site, and methods were collected, therefore allowing us to compare and examine core taxa and previously identified bacterial biomarkers. The samples from the 2021 season were grouped together and differed in the bacterial composition (core taxa), and the biomarkers identified in the first two seasons were absent. Additionally, there was no significant effect of ‘species or ‘organ’ as we had previously observed (Figure 5). Analysis of these samples showed an increased presence of the bacteria *Streptococcus* sp. [mainly specific ASV (ASV0002)] across all the samples (a relative abundance of 10% to 85%) compared to its negligible presence in 2019–2020 seasons (less than 1.5%), which lead to dysbiosis (Tamboli et al., 2004; Egan and Gardiner, 2016). The changes in the relative abundance of *Streptococcus* were also reflected in alpha diversity (Simpson index) and showed that the increase in *Streptococcus* abundance caused a decrease in diversity values and vice versa (Figure 7). This response of decreased bacterial diversity following disease or stress is known in the literature and was also described in humans (Scher et al., 2015). The appearance of *Streptococcus* led to a significant change in the microbiome of the sharks, the relative abundance of certain bacteria was changed, and some bacteria completely disappeared. In addition, to increase in *Streptococcus*, an increase in potential pathogens such as *Photobacterium*, *Vibrio*, and *Acinetobacter* were observed. The observation of a few pathogens appearing together was also described in a wild fish survey in the Mediterranean. The same three bacteria (*Streptococcus*, *Photobacterium*, and *Vibrio* sp.) were detected and appeared, in most cases, in combination together (Meron et al., 2020).

Additional increases in *Pseudomonas* and *Pseudoalteromonas,* previously reported as skin symbionts, were observed mainly in the skin and gills of both shark species and organs. *Pseudoalteromonas* dramatically increased from an average relative abundance of 3% (of all samples), in the first seasons, to 23% in the last season. As described above, this bacterium is an important fish skin symbiont which encodes genes that may promote healthy microbiome-host interactions (Holmström and Kjelleberg, 1999). It is also known to synthesize antimicrobial compounds, which can help in competition with other bacteria (Franks et al., 2006) and inhibit fouling of marine eukaryotes (Bowman, 2007; Doane et al., 2017; Black et al., 2021). Pogoreutz et al. (2019) compared injured and healthy skin of blacktip reef sharks, reporting that *Pseudoalteromonas* was only identified in the sites of injured skin tissue. Based on this observation, along with the increase in potential pathogens seen in our study, it can be hypothesized that the increase in *Pseudoalteromonas* may be a counter-response to the increasing levels of pathogens. This hypothesis may be supported by research showing that *Pseudoalteromonas* species can kill *Vibrio* bacteria by digesting cell walls and subsequently inactivating pathogens (Richards et al., 2017).

The *Streptococcus* sp. genus currently includes >40 recognized species and is cosmopolitan, mainly in animals but also in soil and aquatic environments (Hardie and Whiley, 1997). Some are known to be highly pathogenic, such as the *Streptococcus iniae* and *S. agalactiae* that affect various organs in many fish species worldwide and are linked to warm water (Colorni et al., 2002; Evans et al., 2006; Genin et al., 2020). In marine and freshwater systems, these bacteria cause significant economic losses, estimated at hundreds of millions of dollars each year (Agnew and Barnes, 2007; Klesius et al., 2008; Low et al., 2014). In Israel, two cases of *Streptococcus* infection were reported in the Red Sea. One case was described by Colorni et al. (2002) and was the first report of *S. iniae* in Red Sea fishes. The second reported a massive outbreak of *S. iniae* in 2020 that was very harmful and caused mass mortality of many coral reef fish (Genin et al., 2020). The presence of *Streptococcus* spp. was described in additional cases along the shores of the Mediterranean (Israel). In one case, a moribund wild sandbar shark was found when a post-mortem examination revealed a bacterial infection caused by *S. agalactia* (Morick et al., 2020), while a similar case was recently reported also in a stranded wild common dolphin (Morick et al., 2022). In another study, *S. iniae* was found in the kidneys of several fish species from different geographic locations but, contrary to the previous studies, the fish appeared healthy, and no signs of disease were observed (Meron et al., 2020). Similarly, in our study, no signs of illness or injury were seen in the sharks sampled throughout all three seasons. So far, no information has been published about the health of the sharks in this area, but the high density of the shark (300 m x 150 m estimation) with their exposure to the massive anthropogenic activity (Zemah-Shamir et al., 2019), can enable favorable conditions for the emergence of pathogens and promote a disease outbreak.

### Environmental episode

A comparison between the *Streptococcus* sequences in the NCBI database revealed that our shark sequences (including the dominant ASV0002) were closer to sequences found in terrestrial hosts of Mediterranean origin (Figure 8). Based on this observation and in accordance with previous studies, it can be assumed that the *Streptococcus* spp. identified here may be of terrestrial origin. As described previously, the shark aggregation site is shallow and close to shore. It is where the power and the desalination plant meet and are located near the Hadera stream estuary (Supplementary Figure S1), which is considered a polluted river (Ministry of Environmental Protection, 2020). Thus, the possibility of penetrating terrestrial bacteria (including pathogens) into the marine environment seems possible. This hypothesis was supported by our results showing a similar behavior of the *Streptococcus* in shark and seawater samples. In both, an increasing trend was observed in the presence and relative abundance of the *Streptococcus* sp. at the beginning of the sampling season, its peak in February, and then its decrease (Figures 6, 7). The reflection of this trend may hint at the source of the bacteria and support the overlap we observed between the microbiome of the surrounding seawater and sharks. Examples of similar scenarios, where potential terrestrial pathogens are discovered in marine organisms, have been previously described. For example, *Bartonella* spp., bacteria that are highly adapted to their mammalian reservoir hosts (Boulouis et al., 2005) was first detected in Harbor porpoises (Maggi et al., 2005) and later also in cetaceans and seals (Harms et al., 2008; Morick et al., 2009). In addition, the coccidian protozoan *Toxoplasma gondii* is known to infect many homoeothermic organisms, including humans (Hill and Dubey, 2002). The presence of *T. gondii* has been described in numerous marine mammals around the world (Herder et al., 2015), including a recently reported case from the Mediterranean along the coast of Israel, which identified *T. gondii* in three stranded dolphins (*T. truncates*; Bigal et al., 2018). However, the mechanisms by which these animals are infected remain uncertain. These events highlight the potential damage alien pathogens can inflict on marine organisms and ecosystems.

### Summary

This study summarizes three-time points of sampling (seasons) of the microbiome, which included two shark species and three organs at a site of disturbance along the Israeli coastline. We provided an initial baseline of the sharks’ microbiome, including the core taxa, described its diversity and identified symbiotic and potentially pathogenic bacteria. This information, along with the bacterial biomarkers, will enable future comparison between shark species and organs from different geographic locations and habitats. The changes in the bacterial profile (dysbiosis) detected in the third season emphasize the importance of continuous sampling and including the microbiome aspect in monitoring programs to capture environmental episodes. Microbiome monitoring, besides indicating changes, may assist to identify the type of event (such as specific pollution) and its origin. Our study demonstrated that the microbiome of sharks might serve as an efficient ecologic tool in Long Term Ecological Research (LTER). Additional studies and measures of the sharks (such as blood, hormones, and physiological state) analyzed in tandem with the microbiome will enable a deeper understanding of physiological and environmental changes and support the perception of the meta-organism.

## Data availability statement

The datasets presented in this study can be found in online repositories. The names of the repository/repositories and accession number(s) can be found in the article/Supplementary material.

## Ethics statement

This animal study was authorized with permits issued by Israel Nature and Parks Authority (n. 42412, 42699, 42926).

## Author contributions

GB: conducting the research, analyzing the results, and writing as part of his master’s thesis. DT, AS, and DalM: supervision. ML: data processing and analysis of results, LL, EB, ZZ, DanM, and AS: fieldwork and sampling. AS and EB: project administration. LL, AS, ZZ, DT, and DalM: writing, reviewing, and editing. All authors contributed to the article and approved the submitted version.

## Conflict of interest

The authors declare that the research was conducted in the absence of any commercial or financial relationships that could be construed as a potential conflict of interest.

## Publisher’s note

All claims expressed in this article are solely those of the authors and do not necessarily represent those of their affiliated organizations, or those of the publisher, the editors and the reviewers. Any product that may be evaluated in this article, or claim that may be made by its manufacturer, is not guaranteed or endorsed by the publisher.

## Footnotes

^1^ https://www.nature.com/articles/nmeth.3869

^2^ http://huttenhower.sph.harvard.edu/galaxy

^3^ https://www.arb-silva.de/aligner/
