# Supplementary material for: Preliminary study of shark microbiota at a unique mix-species shark aggregation site, in the Eastern Mediterranean Sea
Source: Front Microbiol. 2023 Feb 23;14:1027804. doi: 10.3389/fmicb.2023.1027804 (PMC9996248; doi:10.3389/fmicb.2023.1027804)
Supplement: Supplementary file 1 [file Data_Sheet_1.PDF]

**Supplementary**

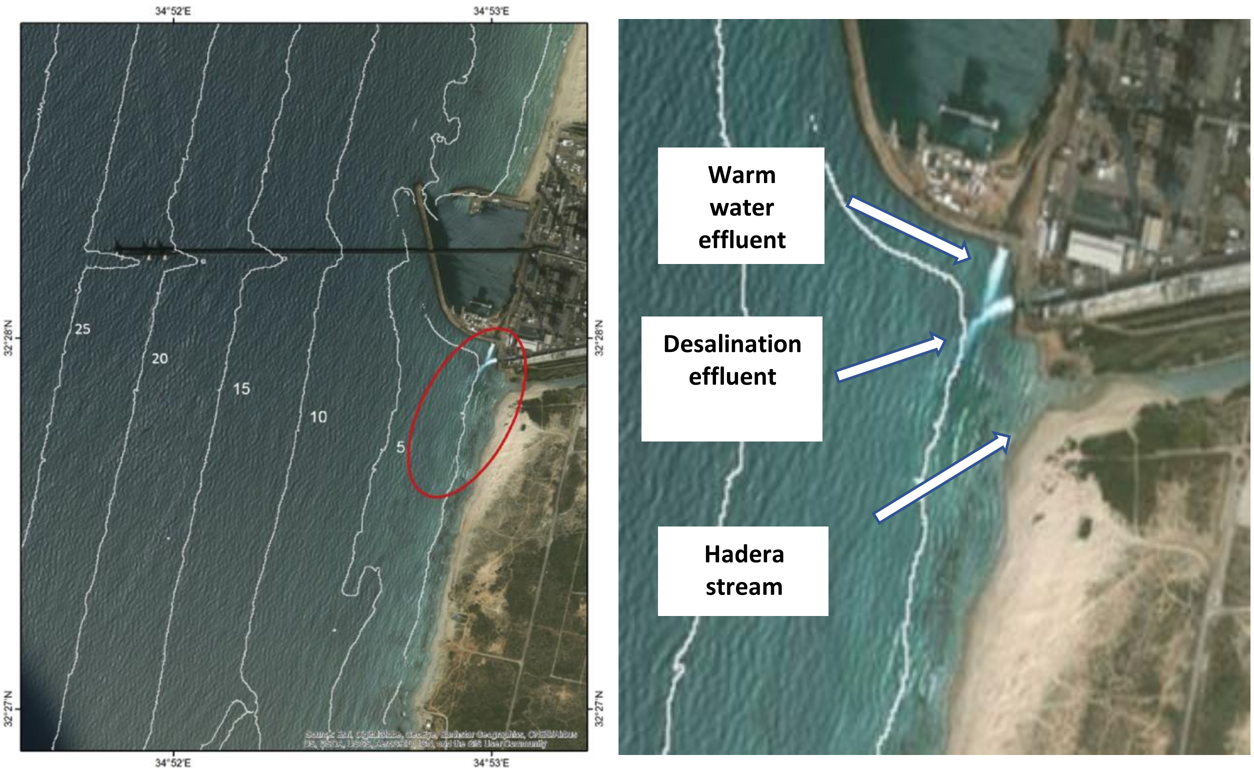


**Figure S1**. Shark aggregation site in Hadera, Israel. Figure adapted from Zemah Shamir et al., 2019.

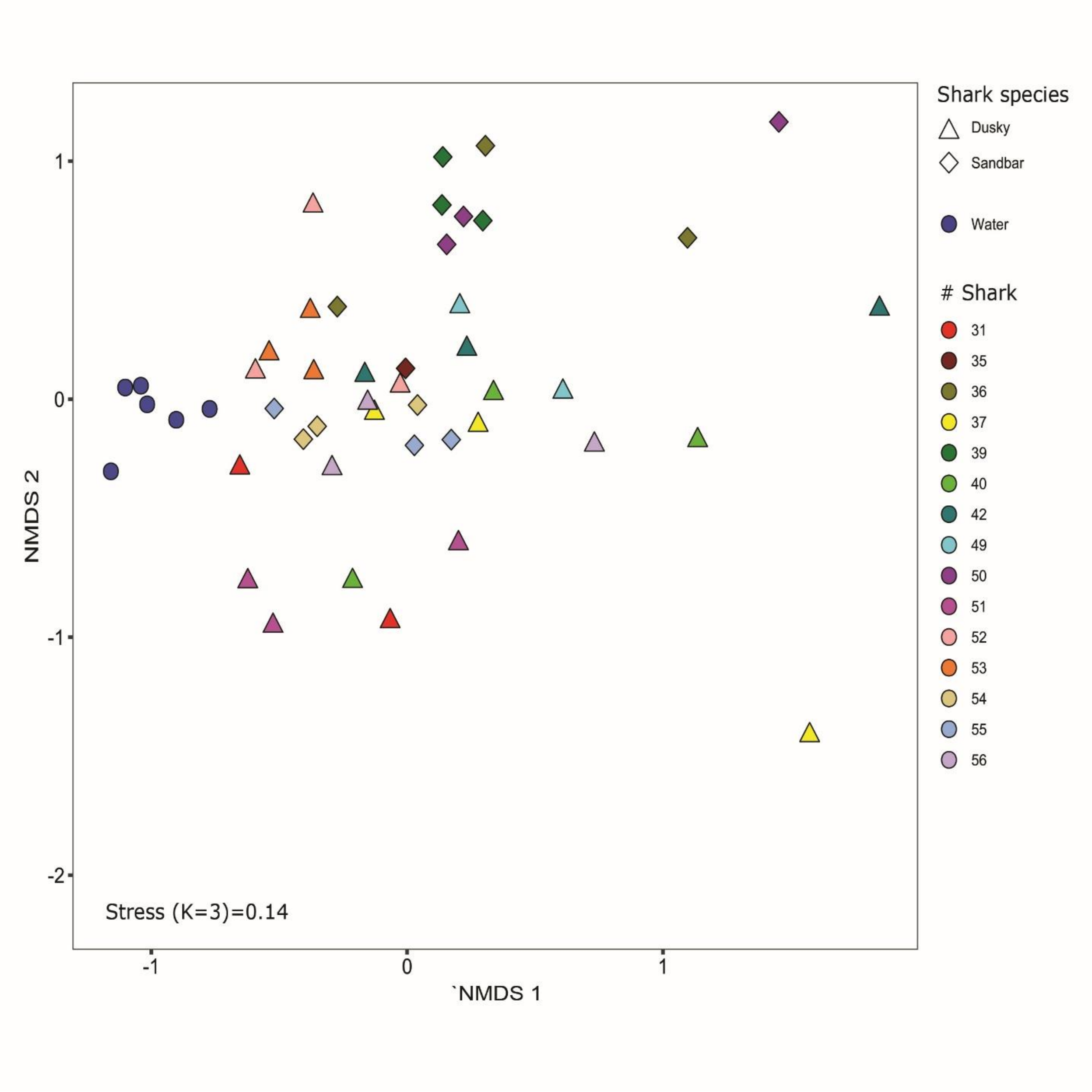


**Figure S2**. Bacterial profiles of shark samples and the surrounding seawater (sampling seasons 2019-2020). Non-metric multidimensional scaling analysis was calculated based on Bray-Curtis dissimilarities among samples. Colors represent the individual sharks, and shapes represent the shark species.

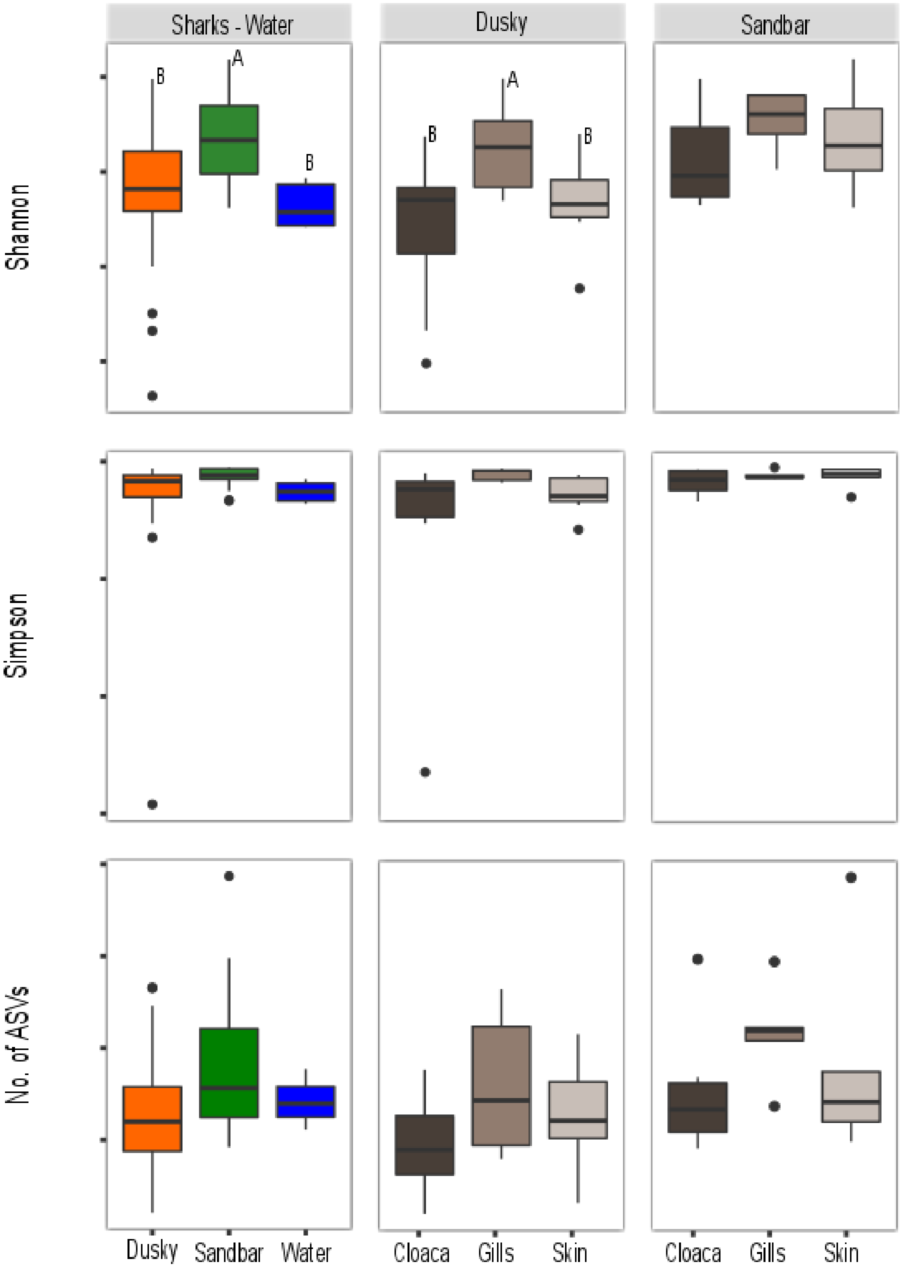


**Figure S3.** Box plots of alpha-diversity indices (Shannon index, Simpson index, and the total number of ASVs). The diversity was compared between shark's species and surrounding seawater and between the organs in each shark species (sampling seasons 2019-2020). The letters A/B represent significant differences.

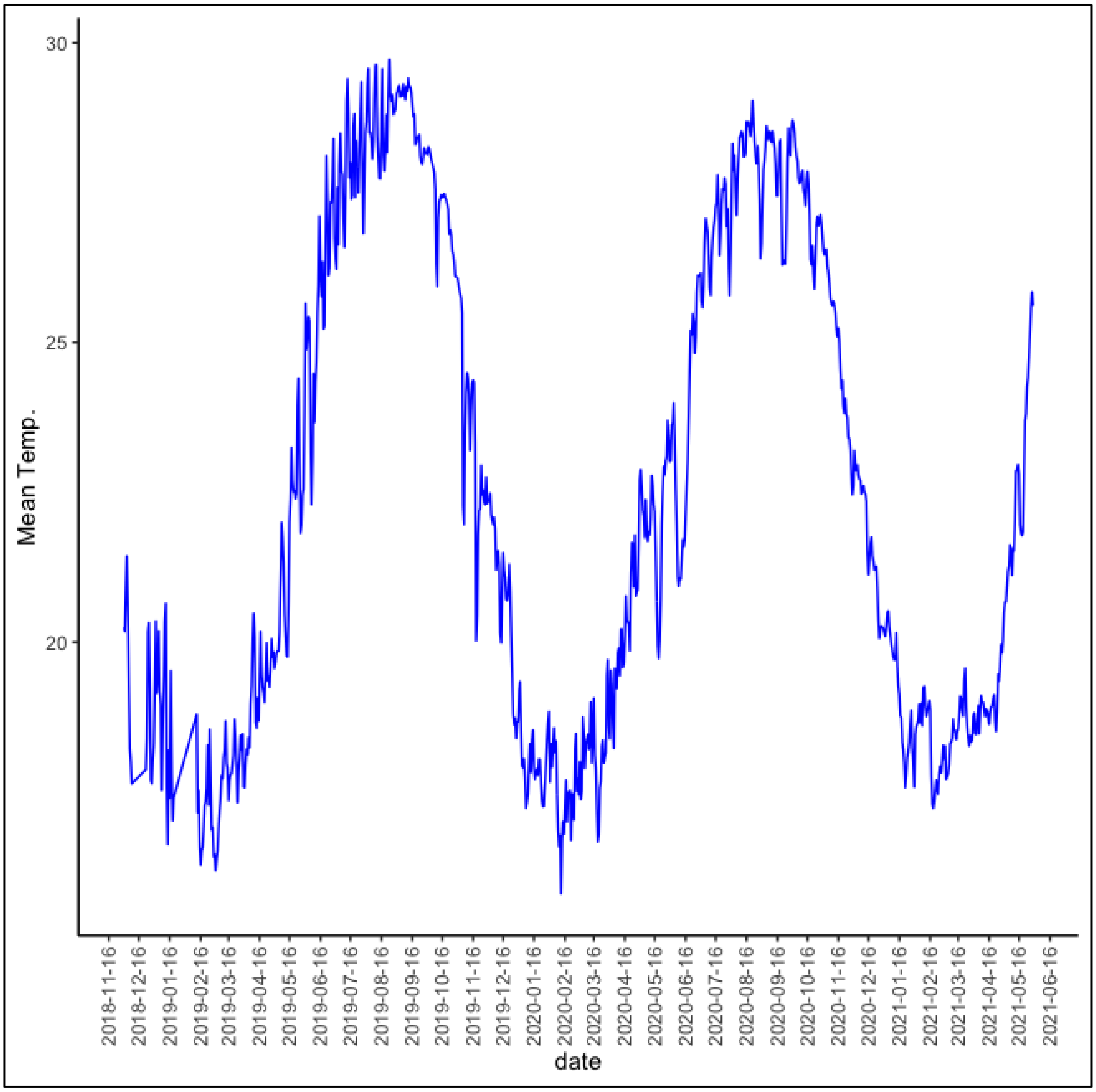


**Figure S4.** Mean ambient seawater temperature around the power and desalination plant's warm water plume. The temperature was measured throughout the research years (2018-2021) in 10 m intervals by four acoustic receivers (Thelma Biotel, Norway) positioned

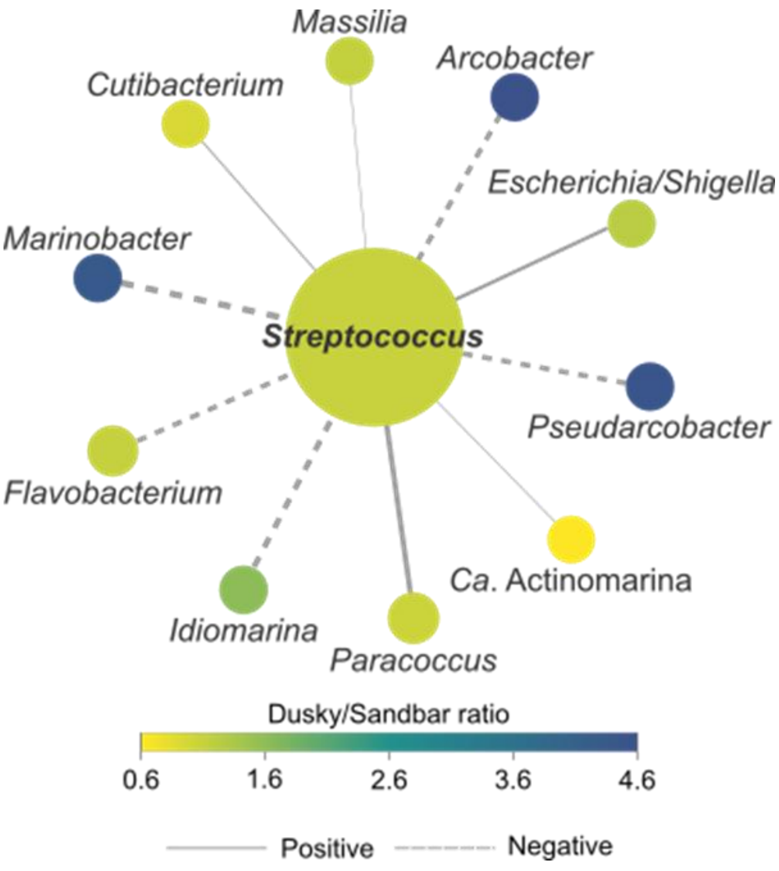


**Fig S5: Co-occurrence of *Streptococcus* and other bacterial genera during 2021 sampling season.** Spearman rho coefficients were calculated between bacterial genera.
Among significant correlations (FDR adjusted P<0.05), 59 correlations were identified between the genus Streptococcus and other genera. Top 5 positive and top 5 negative correlations are presented. Color represents the ratio between relative abundance of the genus in Dusky and Sandbar shark species.

**Table S1.** Summary of all microbiome samples collected over three seasons (**2019-2021**).

| Sampling Season | Test | Number | Cloaca | Gills | Skin |
|---|---|---|---|---|---|
| **2019** (11/2018-5/2019) | Dusky | 5 | 5 | 5 | 4 |
| | Sandbar | 4 | 4 | 3 | 4 |
| | Seawater | 0 | - | - | - |
| **2020** (11/2019-5/2020) | Dusky | 5 | 5 | 5 | 5 |
| | Sandbar | 2 | 2 | 2 | 2 |
| | Seawater | 6 | - | - | - |
| **2021** (11/2020-5/2021) | Dusky | 5 | 5 | 5 | 5 |
| | Sandbar | 6 | 4 | 6 | 6 |
| | Seawater | 6 | - | - | - |

**Table S2:** Effect of species and organ factors on the bacterial composition (sampling seasons 2019-2020). The factors were examined by a pairwise Dunn test. $R^2$ values describe the relative contribution of each factor to variation in microbiota composition are presented. Asterisks represent significant *p*-values ($P<0.05$).

| Test | Test values | | | |
|---|---|---|---|---|
| | **Df** | **$R^2$** | **F** | ***P* value** |
| **Species** | 2 | 0.31 | 11.04 | **0.001***** |
| **Organ** | 2 | 0.06 | 1.99 | **0.018*** |
| Species x Organ | 2 | 0.03 | 1.18 | 0.218 |
| Residual | 43 | | 0.60 | |
| **Shark species / Seawater pairwise** | | | | |
| **Dusky - Sandbar** | 1 | 0.18 | 9.48 | **0.001***** |
| **Dusky - Seawater** | 1 | 0.30 | 13.82 | **0.001***** |
| **Sandbar - Seawater** | 1 | 0.27 | 7.65 | **0.001***** |
| **Organs / Seawater pairwise** | | | | |
| Cloaca - Gills | 1 | 0.03 | 1.00 | 0.399 |
| Cloaca - Skin | 1 | 0.05 | 1.51 | 0.100 |
| **Gills - Skin** | 1 | 0.06 | 1.72 | **0.039*** |
| **Cloaca - Seawater** | 1 | 0.30 | 8.65 | **0.001***** |
| **Gills - Seawater** | 1 | 0.30 | 8.46 | **0.001***** |
| **Skin - Seawater** | 1 | 0.37 | 10.16 | **0.001***** |

## References

Shamir, Z.Z., Shamir, S.Z., Becker, N., Scheinin, A. and Tchernov, D., 2019. Evidence of the impacts of emerging shark tourism in the Mediterranean. *Ocean & Coastal Management*, *178*, p.104847.
